# Network-based pharmacology and UHPLC-Q-Exactive-Orbitrap-MS reveal *Jinhua Qinggan* granule's mechanism in reducing cellular inflammation in COVID-19

**DOI:** 10.3389/fimmu.2024.1382524

**Published:** 2024-07-04

**Authors:** Liping Qian, Zehua Zeng

**Affiliations:** ^1^ Department of Traditional Chinese Medicine, Tsinghua University Hospital, Tsinghua University, Beijing, China; ^2^ School of Chemistry and Biological Engineering, University of Science and Technology Beijing, Beijing, China; ^3^ Daxing Research Institute, University of Science and Technology Beijing, Beijing, China

**Keywords:** *Jinhua Qinggan* granules, traditional Chinese medicine, COVID-19, single-cell RNA sequencing, cellular inflammation

## Abstract

**Introduction:**

The outbreak of SARS-CoV-2, leading to COVID-19, poses a major global health threat. While specific treatments and vaccines are under development, Traditional Chinese Medicine (TCM) has historically been effective against pandemics, including viral pneumonias. Our study explores the efficacy and mechanisms of Jinhua Qinggan Granules (JHQG) in treating COVID-19.

**Methods:**

We analyzed JHQG’s components using UHPLC-Q-Exactive-Orbitrap-MS, identifying 73 compounds. Network pharmacology and single-cell RNA sequencing (scRNA-seq) were used to assess JHQG’s effects on immune cells from peripheral blood mononuclear cells (PBMCs). Literature review supported the antiviral and anti-inflammatory effects of JHQG.

**Results:**

JHQG targets were found to interact with immune cells, including neutrophils, monocytes, plasmablasts, and effector T cells, reducing their overactivation in severe COVID-19. JHQG’s modulation of these cells’ activity likely contributes to reduced inflammation and improved clinical outcomes.

**Discussion:**

Our findings provide insights into JHQG's mechanism of action, highlighting its potential in controlling the inflammatory response in COVID-19 patients. The study supports the use of JHQG as a safe and effective treatment for COVID-19 and similar viral infections, leveraging its ability to modulate immune cell activity and reduce inflammation.

## Main

The COVID-19 pandemic, caused by severe acute respiratory syndrome coronavirus 2 (SARS-CoV-2), was declared official in 2020. Four years on, the global crisis remains unabated (1–3). Infection with SARS-CoV-2 triggers a myriad of immune reactions in the peripheral blood, including heightened pro-inflammatory cytokine levels (4–6), the emergence of inflammatory monocyte subsets (7), lymphopenia (8, 9), T-cell exhaustion (10, 11), and plasma-cell overreactivity (12). Such amplified immune responses can precipitate a cytokine storm, which worsens patient prognoses (13).

After a thorough evaluation, the World Health Organization (WHO) has endorsed Traditional Chinese Medicine (TCM) as a valuable complementary approach for treating mild to moderate cases of COVID-19. TCM has demonstrated efficacy in accelerating viral clearance, alleviating clinical symptoms, and reducing hospitalization durations (14). Similarly, China’s health authorities have sanctioned various TCM treatments for COVID-19 (15). One such therapy, Jinhua Qinggan Granules (JHQG), is advocated for fatigue and fever symptoms in affected individuals (16).

Studies confirm that JHQG not only addresses viral infections but also modulates immune responses, thereby slowing the disease progression (17). In this context, recent clinical investigations have provided evidence supporting JHQG’s properties in reducing cellular inflammation. For instance, a clinical study observed a significant decrease in C-reactive protein (CRP) - a marker of inflammation - following the administration of JHQG in COVID-19 patients, indicating a substantial anti-inflammatory response (P<0.05) (17). Another pivotal aspect of JHQG’s mechanism of action involves the mitigation of the cytokine storm, a severe hyperinflammatory condition associated with COVID-19. A honeysuckle extract component of JHQG was found to significantly reduce cytokine levels (18). Moreover, IL-6, a cytokine critically involved in immune dysregulation leading to cytokine storm, was observed to be significantly reduced in COVID-19 patients treated with JHQG (19). These findings are incrementally establishing JHQG’s role not just in treating viral infection, but importantly, in regulating the immune system response that is critical to patient recovery.

Nevertheless, JHQG’s intricate makeup, comprising 12 distinct medicinal components, including Lonicera japonica Thunb. (Jinyinhua, 金銀花), Gypsum Fibrosum (Shigao, 石膏), Ephedra sinica Stapf (Mahuang, 麻黃), Prunus armeniaca L. (Kuxingren, 苦杏仁), Scutellaria baicalensis Georgi (Huangqin, 黃芩), Forsythia suspensa (Thunb.) Vahl (Lianqiao, 連翹), Fritillaria thunbergii Miq. (Zhebeimu, 浙貝母), Anemarrhena asphodeloides Bunge (Zhimu, 知母), Arctium lappa L. (Niubangzi, 牛蒡子), Artemisia annua L. (Qinghao, 青蒿), Mentha canadensis L. (Bohe, 薄荷), and Glycyrrhiza inflate Batalin (Gancao, 甘草) (20). Although Traditional Network Pharmacology attempts to extract compounds from HERB databases (21), discrepancies between these databases and JHQG’s actual constituents complicate the identification of its anti-inflammatory ingredients.

Addressing this complexity, our investigation involved the analysis of 73 authentic compound components identified in JHQG via the HPLC-Q-Exactive-Orbitrap-MS technique (20). We pinpointed the target proteins associated with these compounds within a comprehensive Network Pharmacological database (21). By leveraging single-cell sequencing data from a COVID-19 patient cohort and healthy individuals (22), we constructed an innovative interaction network connecting herbal compounds, target proteins, and peripheral blood cells. This enabled us to identify monocytes, plasma cells, granulocytes, and effector T cells as pivotal in JHQG’s mitigation of inflammation.

## Results

### Mass spectrometry-based target protein network of the active ingredients in Jinhua Qinggan Granules against COVID-19

**Figure 1:**
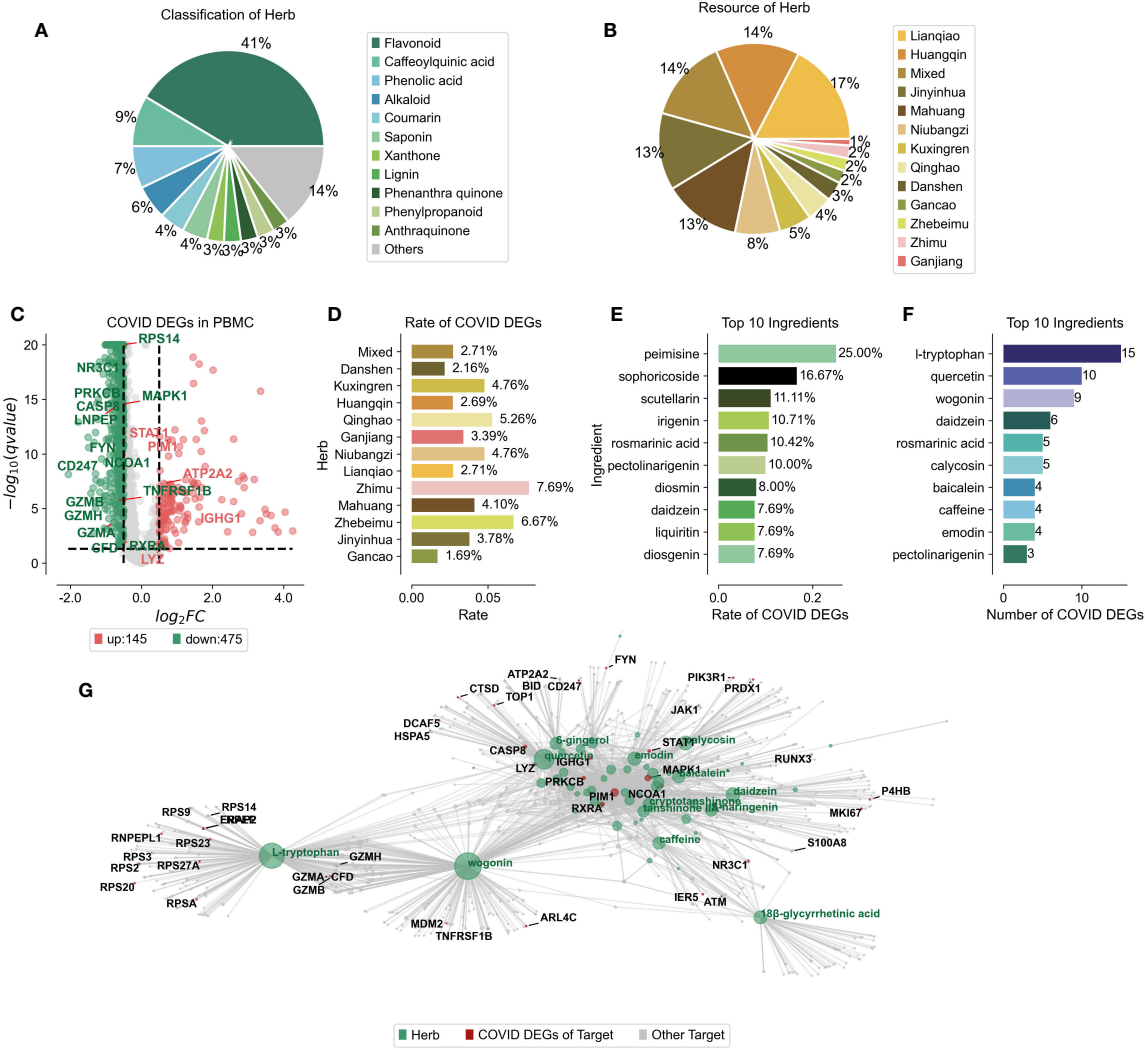
Ingredient Composition and Compound-Target Protein Network of Jinhua Qinggan Granules. **(A)** Percentage of compounds classification in the Chinese herbs of Jinhua Qinggan Granules. **(B)** Percentage of compounds resource in the Jinhua Qinggan Granules. **(C)** Differentially expressed genes (DEGs) in PBMC from COVID-19 patients. Horizontal coordinates represent fold changes of differential expression and vertical coordinates represent two-tailed ttest significance test pvalue. **(D)** Proportion of COVID-19 DEGs in the target proteins of different Chinese herbs in Jinhua Qinggan Granules. **(E)** Proportion of top 10 COVID-19 DEGs in the target proteins of compounds in Jinhua Qinggan Granules. **(F)** Number of top 10 COVID-19 DEGs in the target proteins of compound in Jinhua Qinggan Granules. **(G)** Compound-target protein interactions network of Jinhua Qinggan Granules, where green represents compounds, red represents target proteins differentially expressed at COVID-19, and grey represents other target proteins.

Mass spectrometry has revealed the complex network of target proteins interacting with the active constituents in Jinhua Qinggan Granules (JHQG), offering insights into their therapeutic effects against COVID-19. Recent study identified 73 components within JHQG through mass spectrometry analysis (**Supplementary Table 1**) (20). We standardized the names and classification details of these ingredients using the HERB database. Predominantly, flavonoid analogs represented 41% of these components. Notably, caffeoylquinic acid, phenolic acid, and alkaloids also comprised significant proportions, exceeding 5%, 9%, 7%, and 6%, respectively (**Figure 1A**). We determined the distribution of these compounds across various herbs; for instance, Lianqiao contributed 17%, Huangqin 14%, Jinyinhua 13%, Mahuang 14%, and Niubangzi 8% of the constituents (**Figure 1B**).

To establish a compound-target protein interaction network that could influence serum inflammation in COVID-19 patients, we first analyzed peripheral blood mononuclear cells (PBMCs) from seven hospitalized COVID-19 patients, including four with acute respiratory distress syndrome, and compared them to six healthy controls. We identified 145 up-regulated and 475 down-regulated differentially expressed genes (DEGs) (**Figure 1C**) and linked these to the 73 target proteins associated with the JHQG compounds from the HERB database (**Supplementary Table 2**). The analysis revealed significant DEG representation in proteins associated with herbs such as Zhimu, Zhebeimu, and Qinghao, each exceeding 5% (**Figure 1D**). The top ten compounds implicated in the network—peimisine, sophoricoside, scutellarin, irigenin, rosmarinic acid, pectolinarigenin, diosmin, daidzein, liquiritin, and diosgenin—were also identified based on the percentage of DEGs (**Figure 1E**). Intriguingly, the compounds with the most considerable DEG correspondence—daidzein and pectolinarigenin—were not those with the highest percentage representation alone (**Figure 1F**). a Leveraging the connections between these compounds, target proteins, and DEGs in COVID-19 patients, we constructed a detailed interaction network for JHQG, visualized using plotpy, with an extended view accessible on GitHub (https://starlitnightly.github.io/Analysis_JHQG_COVID/) (**Figure 1G**).

### An Atlas illustrating the impact of Jinhua Qinggan granules on peripheral blood mononuclear cells in COVID-19 patients

**Figure 2:**
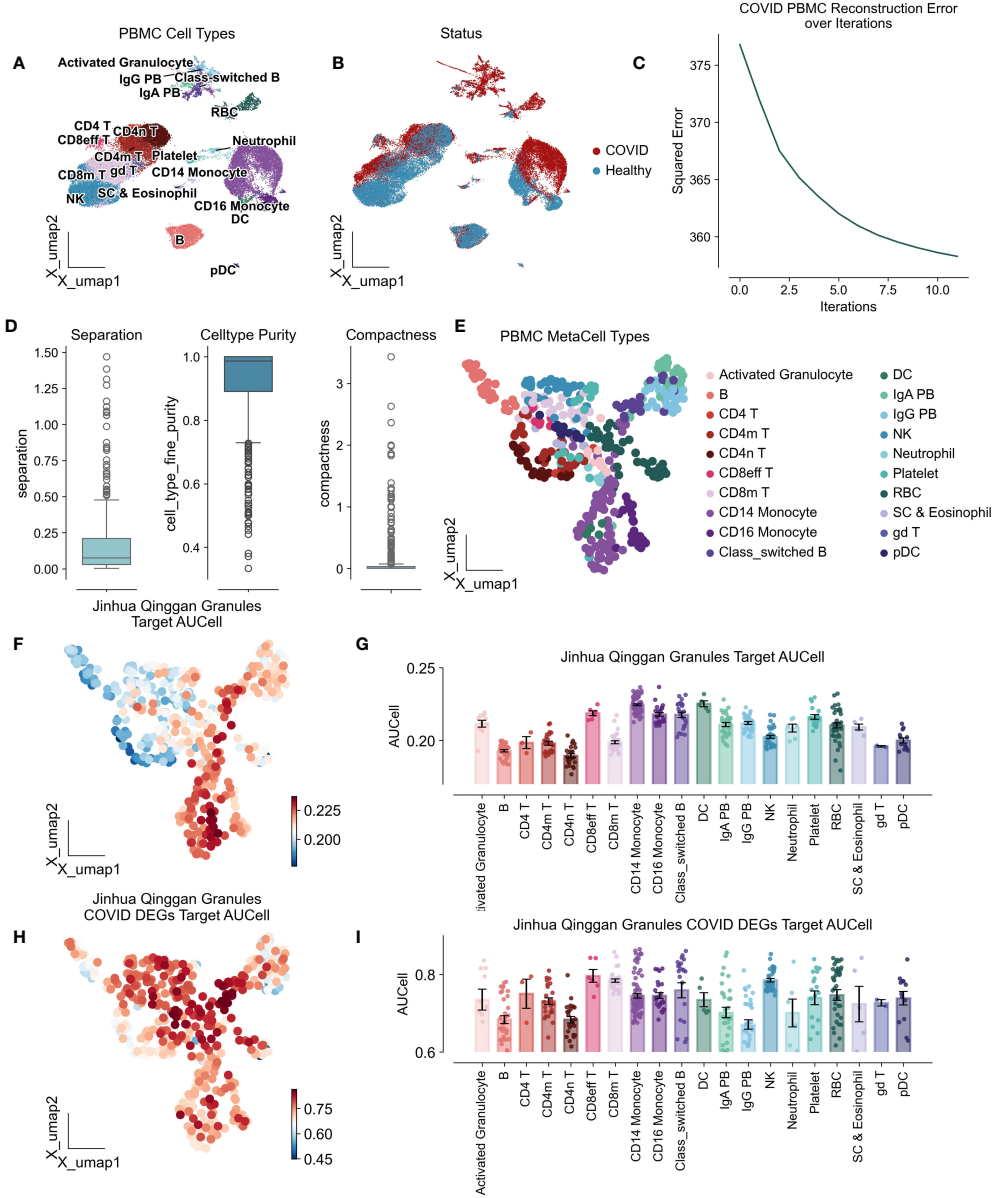
Atlas of peripheral mononuclear cells of COVID-19 by the action of Jinhua Qinggan granules. UMAP plot visualizes scRNA-seq from PBMCs in COVID-19 patients and healthy individuals, colored by cell types **(A)** and COVID status **(B)**. **(C)** Metacellular model iteration loss curves with horizontal coordinates representing the number of iterations and vertical coordinates representing the standard deviation. **(D)** Metacellular quality assessment indicators: metacell separation (distance between nearest metacell neighbor in diffusion space; Methods). Greater separation indicates better performance. metacell compactness (average diffusion component standard deviation; Methods). A lower score indicates more compact metacells. **(E)** UMAPs highlighting metacells of the PBMC in COVID-19 patients and healthy individuals. **(F)** UMAPs plot showing the AUCells score of Jinhua Qinggan Granules’ Target proteins. **(G)** The AUCells score of JHQG’ Target proteins in different cells. **(H)** UMAPs plot showing the AUCells score of JHQG’ Target proteins in COVID-19 DEGs. **(I)** The AUCells score of JHQG’ Target proteins in COVID-19 DEGs in different cells.

To investigate the immunomodulatory effects of JHQG on peripheral blood mononuclear cells (PBMCs) in COVID-19, we conducted an analysis of scRNA-seq data obtained from eight peripheral blood samples collected from seven hospitalized patients and six healthy individuals, as sampled by Wilk et al’s study (22). The seven patients profiled were male, aged 20 to >80 years, healthy controls were asymptomatic, four male and two female, and aged 30–50 years (**Supplementary Table 3**). Following quality control, a total of 44,721 cells were categorized, including activated granulocytes, B cells, various T cell subsets (CD4 naïve, CD4+, CD4 memory, CD8 effector, CD8 memory), monocytes (CD14+ and CD16+), class-switched B cells, dendritic cells (DCs), plasma cells (IgA+ and IgG+), natural killer (NK) cells, neutrophils, platelets, red blood cells (RBCs), stem cells and eosinophils, and gamma delta (γδ) T cells. Of these, 28,094 cells were derived from COVID-19 patients, while 16,627 cells were from healthy controls (**Figures 2A, B**). To enhance the precision and robustness of our analysis, we applied SEACells to calculate 447 metacells from the PBMC data (**Figure 2C**), resulting in metacells with high purity (over 0.9), low separation (below 0.25), and compactness close to zero, indicating successful metacell extraction (**Figures 2D, E**).

Analysis of metacells derived from PBMCs of COVID-19 patients showed that the target proteins associated with JHQG were primarily expressed in monocytes, dendritic cells, and plasma cells (**Figures 2F, G**). Interestingly, upon examining the 45 proteins differentially expressed in COVID-19 against the granules’ target proteins, it became apparent that nearly all cell types were affected by Jinhua Qinggan Granules (**Figures 2H, I**).

### Jinhua Qinggan granules alleviate cellular immune inflammatory networks

**Figure 3:**
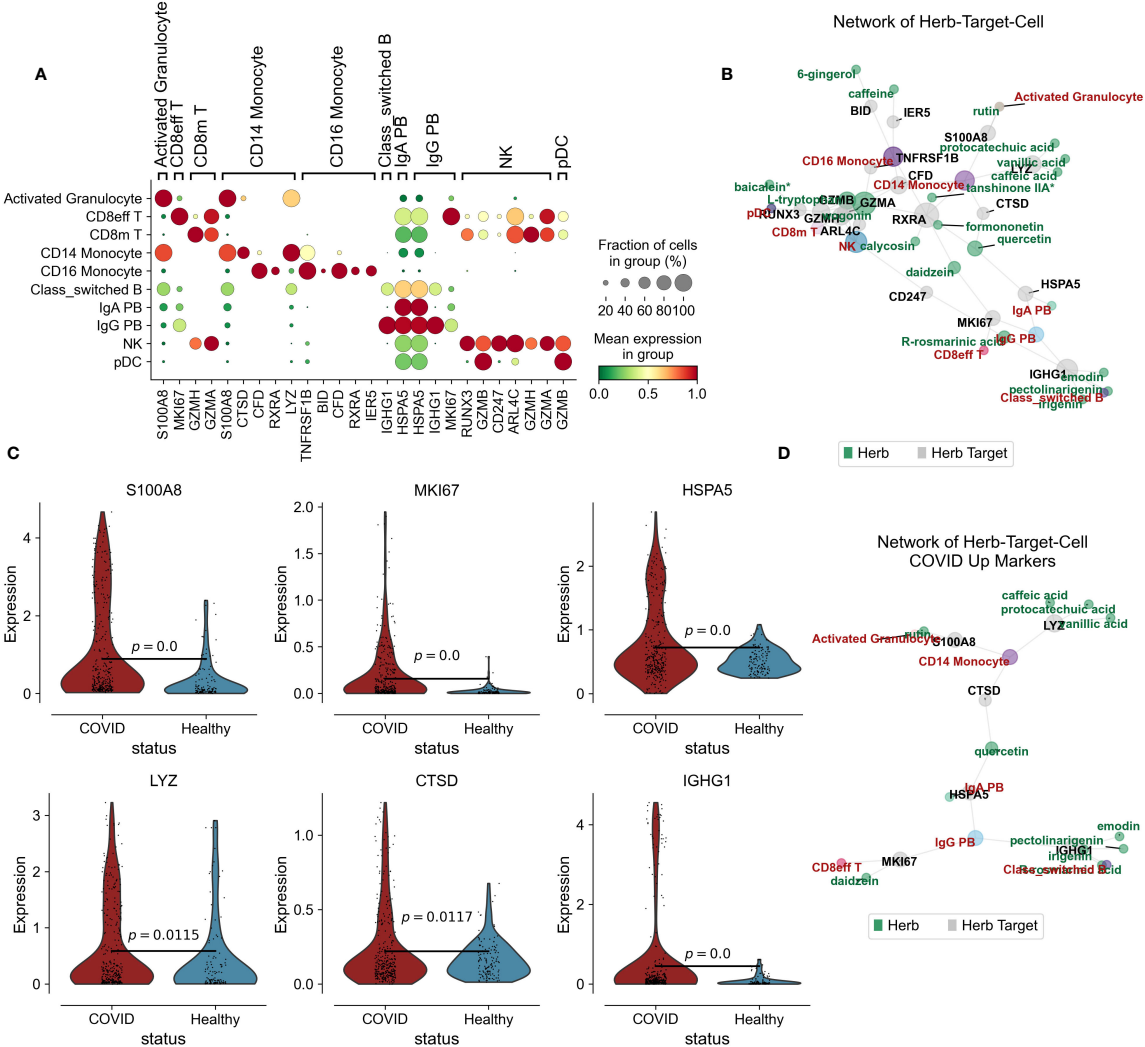
Herb-Target-Cell Network of Jinhua Qinggan Granules. **(A)** Dotplot showing the target marker genes’ mean expression and fraction of cells in group (%). **(B)** Network of Jinhua Qinggan Granule Herb-Target (COVID-19 DEGs)-Cell (PBMC). Grey represent the Herb Target protein in COVID-19 DEGs, Green represent the compound of Jinhua Qinggan Granule, Red represent the target PBMC. **(C)** Violin plot showing the expression of S100A8, MKI67, HSPA5, LYZ, CTSD and IGHG1 in different states of PBMC. **(D)** Network of Herb-Target (COVID-19 Upregulated DEGs)-Cell (PBMC).

In our investigation into the immunomodulatory potential of Jinhua Qinggan Granules in reducing inflammation within peripheral blood, we analyzed the top 20 highly variable genes for each cell type. These findings were cross-referenced with the differentially expressed genes observed in COVID-19 patients and the target genes affected by JHQG. Notably, S100A8, a surface antigen on activated granulocytes, is regulated by rutin, while the proliferation marker MKI67 in CD8+ effector T cells is targeted by daidzein. Additional key genes, such as GZMH in CD8+ memory T cells, CTSD in CD14+ monocytes, RXRA and CFD in CD16+ monocytes, IGHG1 and HSPA5 in class-switched B cells, and CD247 in NK cells, are regulated by various compounds including wogonin, L-tryptophan, quercetin, tanshinone IIA*, formononetin, irigenin, emodin, pectolinarigenin, and calycosin (**Figures 3A, B**).

Increased levels of S100A8, MKI67, HSPA5, LYZ, CTSD, and IGHG1 were observed in COVID-19 patients. However, the interaction with Jinhua Qinggan Granules components led to a downregulation of these genes. Reducing the expression levels of S100A8, LYZ, and CTSD can mitigate the hyperactive immune response from monocytes, diminishing inflammation. Moreover, lower expression of MKI67 can curtail the overactivity of CD8 effector T cells, while reduced HSPA5 and IGHG1 can decrease the inflammatory antibody release from plasma cells (**Figures 3C, D**). Collectively, we constructed a network depicting the interactions among the components of Jinhua Qinggan Granules, COVID-19-specific target proteins, and cellular responses, aiming to reduce excessive inflammatory and immune reactions.

**Figure 4:**
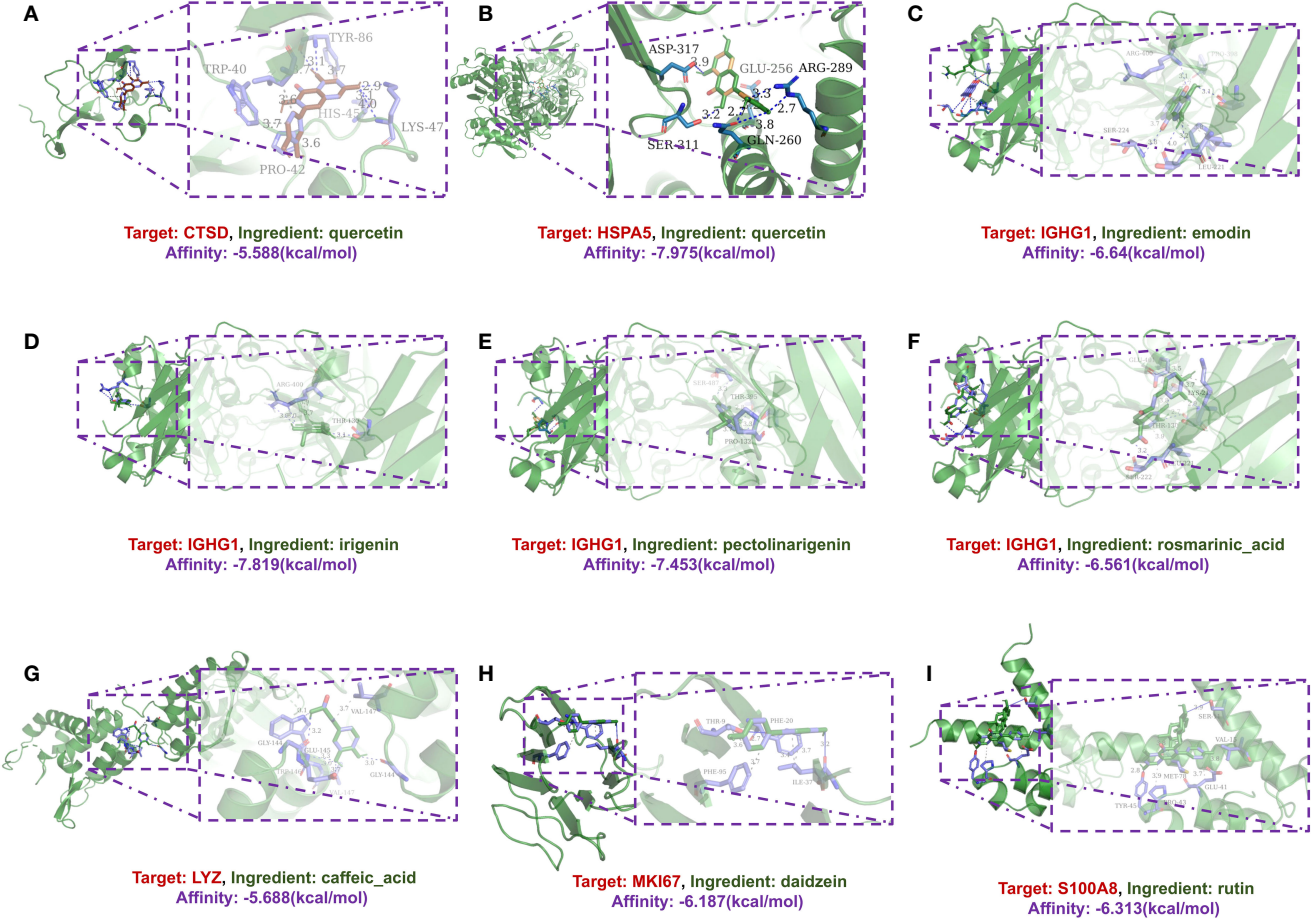
A schematic 3D representation of the molecular docking model. Active sites, binding distances, and ray tracing of compound and target proteins were predicted. **(A)** Quercetin in the protein CTSD (PDB ID: 4OBZ). **(B)** Quercetin in the protein HSPA5 (PDB ID: 3IUC). **(C–F)** Emodin, Irigenin, Pectolinarigenin and Rosmarinic acid in protein IGHG1 (PDB ID: 6HYG). **(G)** Caffeic acid in protein LYZ (PDB ID: 1XJU). **(H)** Daidzein in protein MKI67 (PDB ID: 2AFF). **(I)** Rutin in protein S100A8 (PDB ID: 5HLV).

Additionally, molecular docking simulations were performed to validate the interactions within the network. Among these, the binding affinity of CTSD with quercetin was notable at -5.588 kcal/mol, as was the affinity between HSPA5 and quercetin at -7.975 kcal/mol. Similarly, the binding energies for IGHG1 with different compounds such as emodin, irigenin, pectolinarigenin, and rosmarinic acid showed promising values (-6.64, -7.819, -7.453, and -6.561 kcal/mol, respectively), as did the interaction of LYZ with caffeic acid (-5.688 kcal/mol), MKI67 with daidzein (-5.688 kcal/mol), and S100A8 with rutin (-6.313 kcal/mol) (**Figure 4**).

## Discussion

Traditional Chinese Medicine (TCM) offers a gentler therapeutic approach for COVID-19 patients with varying disease severity, mitigating clinical deterioration (23). This study suggests that early administration of JHQG alleviates viral infection symptoms. Individuals who survived Omicron infection have subjectively reported alleviation of symptoms associated with upper respiratory tract infections, including cough and sore throat, following treatment with JHQG (24). Furthermore, cohort studies demonstrated that the time until viral nucleic acid clearance (test negative) and recovery from pneumonia were significantly shorter in the JHQG group compared to the control group, averaging 10 ± 4 days versus 10 ± 5 days, and 8 ± 4 days versus 10 ± 5 days, respectively (P = 0.010 and 0.021). Moreover, the JHQG group exhibited a significantly higher 7-day viral clearance rate of 56.82% compared to 27.78% in the control group (P = 0.009), with no reported adverse effects of the treatment (25). Overall, these results collectively demonstrate the effectiveness of JHQG in treating COVID-19.

Beyond promoting viral clearance, JHQG also appears to mitigate immune inflammation and reduce the duration of such inflammation in patients (26). However, the mechanisms underlying JHQG’s anti-inflammatory effects and its role in reducing the duration of inflammation remain to be fully elucidated (17). Our study introduces a novel network pharmacological framework that leverages mass spectrometry data on authentic JHQG components, a compound-target database, and single-cell patient data to pinpoint the immune cells that JHQG modulates in peripheral blood. Specifically, we discovered that rutin inhibits activated granulocytes, cells frequently associated with severe COVID-19 progression (27), while monocytes and plasmablasts—which can elicit a strong peripheral humoral response—are also dampened following JHQG administration (27, 28).

Nonetheless, certain limitations in our research warrant mention. The study did not incorporate scRNA-seq from COVID-19 patients treated with authentic JHQG formulations. Instead, potential effector cells were inferred through target protein analysis. As such, we could only deduce cells affected by inhibitory actions of JHQG, not those possibly stimulated by the treatment. Moreover, while the study drew on known compound-protein interactions from the database and utilized Autodock-vina for docking simulations to confirm these relationships, considering protein isoforms and complex cellular contexts, the authenticity of JHQG target proteins demands further experimental validation. These analyses greatly narrowed the scope and cost required for subsequent experimental verification.

In summary, the innovative network we devised, integrating herbs, compounds, target proteins, and cells, offers insights into the network pharmacology of TCM and its therapeutic implications. Notably, our findings demonstrate that JHQG suppresses the activity of activated granulocytes, monocytes, plasmablasts, and effector T cells in COVID-19 peripheral blood, potentially limiting disease progression and diminishing humoral responses and inflammation duration.

## Materials and methods

### Component detection of Jinhua Qinggan granules

The UHPLC-Q-extractive-Orbitrap-MS provided all compound of Jinhua Qinggan Granule (20). Astragalin (kaempferol 3-O-glucoside, Cas. 480–10-4, C21H20O11, 448.38, 97%), oroxin A (baicalein 7-O-glucoside, Cas. 57396–78-8, C21H20O10, 432.38, 97%), mangiferin (Cas. 4773–96-0, C19H18O11, 422.34, 97%), luteolin 7-O-glucuronide (29741–10-4, C21H18O12, 462.36, 97%), ethyl caffeate (Cas. 102–37-4, C11H12O4, 208.12, 97%), scutellarin (Cas. 27740–01-8, C21H18O12, 462.37, 97%), 6-gingerol (Cas. 23513–14-6, C17H26O3, 293.39, 97%), methyl benzoate (Cas. 93–58-3, C8H8O2, 136.148, 97%), rutin (Cas. 153–18-4, C27H30O16, M.W. 610.518, 98%), baicalein (Cas. 491–67-8, C15H10O5, 270.24, 97%), wogonin (Cas. 632–85-9, C16H12O5, 284.26, 97%), chlorogenic acid (Cas. 327–97-9, C16H18O9, M.W. 354.31, 98%), isoquercitrin (Cas. 482–35-9, C21H20O12, M.W. 464.38, 98%), isochlorogenic acid C (4,5-O-dicaffeoylquinic acid, Cas. 57378–72-0, C25H24O12, M.W. 516.45, 98%), vanillic acid (Cas. 121–34-6, C8H8O4, M.W. 168.15, 98%), luteoloside (Cas. 5373–11-5, C21H20O11, M.W. 448.38, 98%), and salidroside (Cas. 10338–51-9, C14H20O7, 300.304, 97%) were obtained from Chengdu Alfa Biotechnology Co., Ltd (Chengdu, China).

18β-Glycyrrhetinic acid (Cas. 471–53-4, C30H46O4, M.W. 4470.7, 98%), cosmosiin (apigenin 7-O-glucoside, Cas. 578–74-5, C21H20O10, 432.4, 98%), licoricesaponin H2 (Cas. 135815–61-1, C42H62O16, 822.9, 98%), liquiritin (Cas. 551–15-5, C21H22O9, 418.4, 98%), quinic acid (Cas. 77–95-2, C7H12O6, M.W. 192.2, 98%), rhein (Cas. 478–43-3, C15H8O6, 284.2, 98%), cryptotanshinone (35825–57-1, C19H20O3, 296.4, 98%), scoparone (Cas. 120–08-1, C11H10O4, 206.2, 98%), and tanshinone IIA (Cas. 568–72-9, C19H18O3, 294.4, 98%) were from Shaanxi Herbest Co. Ltd. (Boji, China).

Acteoside (verbascoside, Cas. 61276–17-3, C29H36O15, M.W. 624.59, 98%), isoviolanthin (Cas. 40788–84-9, C27H30O14, M.W. 578.519, 98%), isoliquiritigenin (Cas. 961–29-5, C15H12O4, M.W. 256.253, 98%), formononetin (Cas. 485–72-3, C16H12O4, M.W. 268.264, 98%), 1,3-O-dicaffeoylquinic acid (Cas. 19870–46-3, C25H24O12, 516.455, 97%), 3,4-dicaffeoylquinic acid (3,4-O-dicaffeoylquinic acid, Cas. 14534–61-3, C25H24O12, 516.455, 97%), pectolinarigenin (Cas. 520–12-7, C17H14O6, 314.29, 97%), neomangiferin (Cas. 64809–67-2, C25H28O16, 584.48, 97%), diosmin (Cas. 520–27-4, C28H32O15, 608.54, 97%), peimisine (ebeiensine, Cas. 19773–24-1, C27H41NO3, 427.629, 98%), solancarpidine (Cas. 126–17-0, C27H43NO2, 413.62, 98%), sophocarpine (13,14-Didehydromatridin-15- one, Cas. 145572–44-7, C15H22N2O, 246.35, 98%), daidzein (Cas. 486–66-8, C15H10O4, 254.24, 97%); calycosin (Cas. 20575–57-9, C16H12O5, 284.27, 97%), scutellarein (Cas. 529–53-3, C15H10O6, 286.24, 97%), 5-O-caffeoylquinic acid (neochlorogenic acid, Cas. 906–33-2, C16H18O9, 354.311, 97%), 4-O-caffeoylquinic acid (cryptochlorogenic acid, Cas. 905–99-7, C16H18O9, 354.311, 97%), and irigenin (548–76-5, C18H16O8, 360.31, 97%) were obtained from Chengdu Biopurify Phytochemicals Ltd. (Chengdu, China).

Chrysin (Cas. 480–40-0, C15H10O4, M.W. 254.24, 98%), viscidulin I (Cas. 92519–95-4, C15H10O7, M.W. 302.24, 98%), 2′,6′-dihydroxypinobanksin (Cas. 80366–15-0, C15H12O7, 304.24, 98%), sophoricoside (Cas. 152–95-4, C21H20O10, 432.38, 98%), isorhamnetin-3-O-β-D-glucoside (Cas. 5041–82-7, C22H22O12, 478.4, 98%), 6-prenylapigenin (Cas. 68097–13-2, C20H18O5, 338.36, 98%), forsythoside B (Cas. 81525–13-5, C34H44O19, 756.7, 98%), dalbergioidin (Cas. 30368–42-4, C15H12O6, 288.65, 98%), (−)-epipinoresinol (Cas. 10061–38-8, C20H22O6, 358.39, 96%), and (+)-epipinoresinol (Cas. 24404–50-0, C20H22O6, 358.39, 96%) were purchased from BioBioPha Co., Ltd. (Kunming, China).

Esculetin (Cas. 305–01-1, C9H6O4, M.W. 178. 41, 98%), scopoletin (Cas. 92–61-5, C10H8O4, M.W. 192.17, 98%), vitexin (Cas. 3681–93-4, C21H20O10, M.W. 432.10, 98%), and isoschaftoside (apigenin-6-arabinoside-8-glucoside, Cas. 52012–29-0, C26H28O14, M.W. 564.49, 98%), quercetin (Cas. 117–39-5, C15H10O7, M.W. 302.23, 98%), S-naringenin (Cas. 480–41-1, C15H12O5, M.W. 272.25, 98%), vicenin-2 (Cas. 23666–13-9, C27H30O15, M.W. 594.518, 98%), and schaftoside (apigenin-6-glucoside-8-arabinoside, Cas. 51938–32-0, C26H28O14, M.W. 564.49, 98%) were purchased from Sichuan Weikeqi Biological Technology Co., Ltd. (Chengdu, China).

Chloesteryl acetate (Cas. 604–35-3, C29H48O2, 428.69, 97%) and protocatechuic acid (Cas. 99–50-3, C7H6O4, 154.12, 97%) were form Sigma–Aldrich (Shanghai, China); Caffeic acid (Cas. 331–39-5, C9H8O4, 97%) and emodin (Cas. 518–82-1, C15H10O5, M.W. 270.24, 97%) were obtained from the National Institute for the Control of Pharmaceutical and Biological Products (Beijing, China). D-Gluconic acid (Cas. 526–95-4, C6H11O7, M.W. 195.15, 98%) was from TCI Chemical Co. (Shanghai, China). R-rosmarinic acid (Cas. 20283–92-5, C18H16O8, M.W. 360.3, 98%) and ferulic acid (Cas. 1135–24-6, C10H10O4, M.W. 194.19, 98%) were purchased from Aladdin Chemistry Co. (Shanghai, China). L-Tryptophan (Cas. 73–22-3, C11H12N2O2, M.W. 204.23, 98%) was from J&K Scientific Co., Ltd. (Beijing, China). Danshensu (Cas. 76822–21-4, C9H10O5, M.W. 198.17, 97%) was from Shanghai Acmec Biochemical Co., Ltd (Shanghai, China). Caffeine (Cas. 58–08-2, C8H10N4O2, M.W. 194.191, 98%) was prepared using sublimation method from Green tea [9]. Methanol and water were of mass spectra purity grade.

### Intregient-target protein network construction of Jinhua Qinggan Granules’s compound

We retrieved target proteins from the HERB database (21) (http://herb.ac.cn/) based on the Ingredient Name of the compounds in 73, and a list of database-supported as well as literature-supported target proteins is included.

We then used the plot_network function in omicverse (29) to visualize the 73 components and for that matter the target proteins.

### Analysis of differentially expressed genes in peripheral blood of patients with COVID-19

We first selected annotated single-cell sequencing data from the public database COVID-19 Cell Atlas (https://www.covid19cellatlas.org/#wilk20) for eight peripheral blood samples from seven COVID-19 hospitalized patients and six peripheral blood samples from six healthy individuals. The cohort encompassed seven male patients ranging in age from 20 to over 80 years. Samples were obtained between 2 and 16 days post symptom onset. In contrast, healthy controls, comprising four males and two females, were asymptomatic individuals aged between 30 and 50 years. Half of the eight COVID-19 specimens came from mechanically ventilated patients diagnosed with acute respiratory distress syndrome (ARDS). Distinctively, patient C1 provided two samples: the first at nine days after exhibiting symptoms, at which time he required supplemental oxygen, and a subsequent sample was taken two days later post-intubation. Remdesivir treatment in the hospital setting was given to five patients, with four receiving it before their samples were collected (**Supplementary Table 3**).

Raw sequencing data are available at NCBI Gene Expression Omnibus (accession number GSE150728). Cells with less than 1,000 UMI or more than 15,000 UMI, as well as cells containing more than 20% of reads for mitochondrial genes or rRNA genes (RNA18S5 or RNA28S5), were considered low quality and excluded from further analysis. To remove putative multiplex states (where there may be multiple cells loaded into a given well on the array), cells expressing more than 75 genes per 100 UMI were also filtered out. Genes expressed in fewer than 10 cells were removed from the final count matrix. There were 44,721 cells after quality control.

We then extracted 447 high-quality metacells using the SEACells (30) module in omicverse and performed compaction and segregation assessments. Given that metacells represent distinct cell states of the biological system under consideration, inferred metacells should (1) be compact, meaning that they exhibit low variability among aggregated cells and that most of this variability is a result of measurement noise, and (2) be well separated from neighboring metacells.

We then used the pyDEG module in omicverse to analyse the differential expression of two different status metacells, Contrl and COVID-19, and the significance was calculated using the ttest model. We finally selected differential genes with a differential expression multiplicity of 0.5 in the threshold and an ADJUST p-value of less than 0.05.

### Intregient-target-cell network construction of Jinhua Qinggan Granules’s compound

To construct the Intregient-Target-Cell network, we first used omicverse’s get_celltype_marker function to obtain the marker genes for each cell type for scRNA-seq of COVID-19 peripheral blood samples. Then we took the intersection of COVID-19 differentially expressed genes, cell-specific marker genes and target proteins of JHQG to obtain the cell-specific target genes of COVID-19 differences of JHQG. Subsequently still the plot network function of omicverse was used to draw the Intregient-Target-Cell network for visualization.

### Molecular docking

2D structures of HE obtained from the PubChem database were downloaded as SDF files and then imported into chem3D software to generate their respective free energy-minimized 3D conformations. Additionally, crystal structures of hub genes’ proteins were retrieved from the RCSB Protein Data Bank (https://www.rcsb.org/). The ADFR Suite was utilized to eliminate water molecules and ligands from protein structures, followed by addition of non-polar hydrogens and conversion to PDBQT format (31). Additionally, small molecule ligands (HE) were converted to PDBQT format for docking using the Meeko python package (https://github.com/forlilab/Meeko.git). The protein receptor structure was displayed in secondary structure representation without lines. The active pocket location was determined using AutoGrid4 (32). Subsequently, protein-ligand docking was conducted using Autodock Vina 4.0 software, with lower binding energy indicating greater stability. Ligand-receptor interactions, such as π stacking (parallel and perpendicular), π-cation interactions, hydrogen bonding, water bridges, and salt bridges, were visualized using the Protein-Ligand Interaction Profiler (PLIP) website (https://plip-tool.biotec.tu-dresden.de/plip-web/plip/index) (33).

## Data availability statement

The datasets presented in this study can be found in online repositories. The names of the repository/repositories and accession number(s) can be found in the article/**Supplementary material**. The original codes presented in the study are publicly available. This data can be found here: https://github.com/Starlitnightly/Analysis_JHQG_COVID.

## Author contributions

LQ: Conceptualization, Formal analysis, Investigation, Software, Writing – original draft. ZZ: Conceptualization, Data curation, Formal analysis, Investigation, Methodology, Software, Visualization, Writing – review & editing.
